# HGA Triggers SAA Aggregation and Accelerates Fibril Formation in the C20/A4 Alkaptonuria Cell Model

**DOI:** 10.3390/cells13171501

**Published:** 2024-09-07

**Authors:** Pierfrancesco Mastroeni, Alfonso Trezza, Michela Geminiani, Luisa Frusciante, Anna Visibelli, Annalisa Santucci

**Affiliations:** 1 ONE-HEALTH Lab, Department of Biotechnology, Chemistry and Pharmacy, University of Siena Via Aldo Moro, 53100 Siena, Italy; p.mastroeni@student.unisi.it (P.M.); alfonso.trezza2@unisi.it (A.T.); luisa.frusciante@unisi.it (L.F.); anna.visibelli2@unisi.it (A.V.); annalisa.santucci@unisi.it (A.S.); 2 MetabERN, Department of Biotechnology, Chemistry and Pharmacy, University of Siena Via Aldo Moro, 53100 Siena, Italy

**Keywords:** alkaptonuria, secondary amyloidosis, amyloid, metabolic disease, serum amyloid A, serum amyloid P, HGA, molecular modeling, docking and molecular dynamics simulation

## Abstract

Alkaptonuria (AKU) is a rare autosomal recessive metabolic disorder caused by mutations in the homogentisate 1,2-dioxygenase (HGD) gene, leading to the accumulation of homogentisic acid (HGA), causing severe inflammatory conditions. Recently, the presence of serum amyloid A (SAA) has been reported in AKU tissues, classifying AKU as novel secondary amyloidosis; AA amyloidosis is characterized by the extracellular tissue deposition of fibrils composed of fragments of SAA. AA amyloidosis may complicate several chronic inflammatory conditions, like rheumatoid arthritis, ankylosing spondylitis, inflammatory bowel disease, chronic infections, neoplasms, etc. Treatments of AA amyloidosis relieve inflammatory disorders by reducing SAA concentrations; however, no definitive therapy is currently available. SAA regulation is a crucial step to improve AA secondary amyloidosis treatments. Here, applying a comprehensive in vitro and in silico approach, we provided evidence that HGA is a disruptor modulator of SAA, able to enhance its polymerization, fibril formation, and aggregation upon SAA/SAP colocalization. In silico studies deeply dissected the SAA misfolding molecular pathway and SAA/HGA binding, suggesting novel molecular insights about it. Our results could represent an important starting point for identifying novel therapeutic strategies in AKU and AA secondary amyloidosis-related diseases.

## 1. Introduction 

Alkaptonuria (AKU) is a rare genetic disorder that results from a defect in the metabolism of the amino acids phenylalanine and tyrosine [1]. Specifically, AKU is caused by mutations in the homogentisate 1,2-dioxygenase (HGD) gene, leading to a deficiency of the HGD enzyme [2]. This enzyme deficiency prevents the normal breakdown of homogentisic acid (HGA), causing it to accumulate in the body [1]. The excess HGA is excreted in the urine, which darkens upon exposure to air, a distinctive symptom of the disease [3].

Beyond the urinary symptoms, the chronic buildup of HGA in connective tissues leads to ochronosis, characterized by a bluish-black pigmentation of cartilage and other tissues [4]. This can cause a range of complications, including early-onset osteoarthritis, particularly in the spine and large joints, and potentially heart and kidney issues [2]. Alkaptonuria is a lifelong condition that typically manifests in early childhood but becomes more problematic with age as the cumulative effects of HGA deposition take their toll [5].

A further complication of AKU is caused by the presence of unidentified proteins which are involved in both intra and extracellular ochronotic pigment deposition [6].

Previous studies showed that the AKU disease is also characterized by the presence of serum amyloid A (SAA), classifying AKU as a secondary (AA) amyloidosis [7].

Serum amyloid A is a group of apolipoproteins primarily produced by the liver which binds with high-density lipoprotein (HDL) and plays several critical roles in the immune response. These include facilitating the transport of cholesterol, promoting chemotaxis (the movement of immune cells toward sites of inflammation), and modulating the inflammatory response. These proteins are classified as acute-phase reactants due to their significant upregulation in response to inflammation. During inflammatory events, such as infections, trauma, or acute inflammatory diseases, the concentration of SAA in the bloodstream can increase dramatically, often by up to 1000-fold [8].

Persistently elevated levels of SAA due to ongoing inflammation lead to the misfolding and aggregation of this protein into insoluble amyloid fibrils. These fibrils deposit in various organs and tissues, including the kidneys, liver, spleen, and gastrointestinal tract, impairing their normal function and leading to a pathological condition known as AA secondary amyloidosis [9].

Secondary (AA) amyloidosis, different from primary amyloidosis (which is associated with light chain proteins), is a systemic disorder characterized by the extracellular deposition of amyloid fibrils derived from SAA protein. This condition is a complication of chronic inflammatory diseases, chronic infections, or certain malignancies [10].

The symptoms of secondary amyloidosis are diverse and depend on the organs involved. Common signs include fatigue, weight loss, and swelling due to kidney dysfunction. Gastrointestinal involvement can lead to malabsorption and bleeding, while liver and spleen involvement may cause hepatomegaly and splenomegaly, respectively [11].

Currently, the development of secondary (AA) amyloidosis by persistently high plasma concentrations of SAA is still not clear; however, there is strong evidence that the onset depends on a seeding mechanism exerted by misfolded and aggregated AA protein [12,13].

The full-length SAA1 protein is made of 122 residues, where the first 18 residues represent the signal peptide, which forms the mature SAA1 (104 residues) following cleavage [14]. In the onset of AA amyloidosis, the SAA1 is frequently cleaved, producing different forms of SAA1 fragments [15].

The SAA1 N-terminal region plays a critical role in the formation of amyloidogenic SAA1, as following a misfolding process, it starts SAA1 polymerization and fibril formation. The deletion of a single N-terminal amino acid (Arg-1) has been shown to trigger the formation of SAA1 fibrils and amyloid deposits [16]. Also, the deletion of the C-terminal tail destabilized the SAA1 peptide, triggering fibril formation [17].

Several proteolytic enzymes have been implicated both in the formation and degradation of AA amyloid, like the lysosomal protease cathepsin B, which produces a plethora of fragments, and also metalloproteinases (MMP1, MMP2, and MMP3) are able to degrade the SAA1 into peptides corresponding to the AA protein [18,19].

Despite the numerous SAA1 physiological and structural information and its involvement in fibril formation, several issues remain to be addressed.

In this study, using the HGA-supplemented C20/A4 cellular model (human chondrocytes), we analyzed the amyloidogenic mechanisms in AKU. In vitro evidence showed that the SAA/HGA relationship caused fibril aggregation and formation. In addition, our results suggested an increase in the colocalization between the SAA1 and serum amyloid P (SAP) proteins in cells treated with HGA, indicating their involvement in amyloid fibril formation.

In silico methods showed the ability of HGA to bind on SAA1, causing its structural destabilization and a rapid misfolding of the N-terminal region, similar to the SAA1-ΔArg-1 mutant.

Here, we propose HGA as a small molecule disruptor of SAA1, enhancing in vitro amyloid aggregation, thus showing a key role in AKU amyloidosis. Understanding SAA’s regulations is essential for comprehending its role both in normal immune processes and pathological conditions. Research into SAA1 continues to provide insights regarding its potential role as a biomarker for inflammatory diseases and its involvement in disorder progression, like in AKU, offering prospects for a wide range of targeted therapeutic strategies and precision medicine approaches.

## 2. Materials and Methods

### 2.1. In Vitro Methods

#### 2.1.1. Cells Cultures and Treatment

The C20/A4 human chondrocyte cell line (Sigma-Aldrich, SCC041) was grown in Dulbecco’s modified Eagle’s medium (DMEM, Merck, Darmstadt, Germany) with 1% penicillin/streptomycin (P/S, Merck, Darmstadt, Germany) and 10% fetal bovine serum (FBS, Merck, Darmstadt, Germany) at 37 °C in a humidified environment with 5% CO_2_. HGA (Sigma-Aldrich, St. Louis, MO, USA) was prepared by dissolving it in deionized water to produce a 10 mM stock solution. The initial solution was then diluted in the cell culture medium to achieve concentrations of 0.066 mM and 0.1 mM.

#### 2.1.2. Immunofluorescence Study

C20/A4 human chondrocytes were cultured on glass coverslips with a concentration of 2 × 10^4^ cells for one week and 1 × 10^4^ cells for two weeks. Following the treatments with 0.066 and 0.1 mM of HGA, the cells were fixed with methanol before being permeabilized using pure acetone and then blocked. Next, the cells were incubated overnight at 4 °C with an anti-SAA mouse monoclonal antibody (1:100, Sigma-Aldrich, St. Louis, MO, USA), and an anti-SAP rabbit monoclonal antibody (1:30, Sigma-Aldrich, St. Louis, MO, USA). Afterward, the cells were incubated with Alexa Fluor™ 546 goat anti-mouse Ig (1:100, Thermo Fisher Scientific, Rockford, IL, USA) and Alexa Fluor™ 488 goat anti-rabbit Ig (1:100, Thermo Fisher Scientific, Rockford, IL, USA). Ultimately, the samples were mounted using a fluoroshield mounting medium containing DAPI (Abcam, Cambridge, UK). Images were captured with a fluorescence microscope (Zeiss AxioLabA1, Oberkochen, Germany) and fluorescence intensity was quantified using ImageJ software (Version 1.54). A quantitative colocalization and correlation study of SAP and SAA was conducted using ImageJ and the JACoP plug-in (v2.0). The Manders’ coefficient, which indicates the amount of SAP-overlapping SAA pixels, was calculated. The colocalization plug-in in ImageJ was used to visualize colocalization occurrences as white dots.

#### 2.1.3. In Situ Proximity Ligation Assay (PLA)

In order to assess the colocalization between SAA and SAP, the in situ proximity ligation assay (Duolink kit, Sigma-Aldrich, St. Louis, MO, USA) was conducted following the instructions provided by the manufacturer. In brief, the C20/A4 human chondrocytes were placed in a sterile 24-well plate at 1 × 10^4^ cells/well. The cells were allowed to grow until they reached 80–85% confluence. Then, they were treated with 0.066 mM and 0.1 mM of HGA. Following treatment, the cells were fixed, blocked, and incubated with primary antibodies that specifically target the proteins of interest, following the previously described protocol. Next, the cells were incubated with secondary antibodies conjugated with oligonucleotides (mouse PLUS and rabbit MINUS probes). After the oligonucleotides were hybridized and ligated, DNA amplification was carried out by adding an amplification solution along with polymerase. The products of the PLA detection reaction were visualized as red fluorescent dots. Finally, the cells were mounted using Duolink in situ mounting with DAPI (Sigma-Aldrich, St. Louis, MO, USA) and examined under a fluorescence microscope (Zeiss Axio-LabA1, Oberkochen, Germany).

#### 2.1.4. Detection of Amyloid Deposits

To investigate the distribution of amyloidosis, C20/A4 human chondrocytes were placed into 6-well plates at a concentration of 2 × 10^5^ cells per well and cultured until they reached 80–85% confluence before being treated with HGA at varying concentrations (0.066 mM, 0.1 mM) for 14 days. Cells were then stained with 1% Congo red and dehydrated using ethanol before being examined under a bright-field microscope. A Zeiss Axio Lab A1 microscope was then used to examine the samples under polarized light.

#### 2.1.5. Western Blotting

C20/A4 human chondrocytes at 75–80% confluence were treated with HGA at different concentrations (0.066 mM and 0.1 mM) for two weeks. Following treatment, cells were lysed with RIPA buffer supplemented with phosphate and protease inhibitors and then disrupted by sonication for 5 min. Protein concentration was evaluated according to Bradford. A total of 20 μg of cell protein lysate was resolved by 8% sodium dodecyl sulphate-polyacrylamide gel electrophoresis (SDS-PAGE) and then electrotransferred onto a nitrocellulose membrane (0.45 mm pore size; Cytiva, Marlborough, MA, USA). After blocking for 2 h with 5% non-fat dry milk in phosphate-buffered saline buffer (PBS) at room temperature, membranes were incubated with an anti-SAA rabbit polyclonal antibody (1:1500, Thermo Fisher Scientific, Rockford, IL, USA) and an anti-actin mouse monoclonal antibody (1:4000, Sigma-Aldrich, St. Louis, MO, USA) at 4 °C overnight. The membranes were then incubated with anti-rabbit and anti-mouse HRP-conjugated secondary antibodies (1:80.000, Sigma-Aldrich, St. Louis, MO, USA). Immunoreactive bands were revealed with Luminata Crescendo (Merck Millipore, Burlington, MA, USA) and images were acquired using Image-Quant LAS4000 (GE Healthcare, Milano, Italy). The optical densities of the immunoreactive bands were analyzed using ImageQuantTL software V 7.0 (GE Healthcare, Milano, Italy). Protein levels were normalized against actin, which served as a protein-loading control.

#### 2.1.6. Statistical Analysis

The results are provided from three independent experiments. Statistical analyses were carried out with GraphPad Prism 9.0 software (GraphPad Software, San Diego, CA, USA). The data are expressed as mean ± standard deviation (SD) and were compared using one-way analysis of variance (ANOVA) followed by a post hoc test. A *p*-value of 0.05 or less was considered statistically significant.

### 2.2. In Silico Methods

The primary structure of the human serum amyloid A-1 protein (SAA1) was retrieved from the UniProtKB reviewed (Swiss-Prot) database [20] with P0DJI8 as the entry code. The human apo-state serum amyloid A1 3D structure obtained using an X-ray method with resolution 2.19 Å (PDB ID: 4IP8) was downloaded from the RCSB PDB database [21].

To avoid errors during the molecular dynamic (MD) simulations, potential 3D structure-missing amino acid side chains were added using PyMOD3.0 (Department of Biochemical Sciences, Sapienza University, Rome, Italy) [22] and validated through PROCHECK [23]. Potential steric clashes were solved using energy minimization and the structure was relaxed using a short MD run. Thus, the CHARMM-GUI platform (Lehigh University, Bethlehem, Pennsylvania, USA) [24] was used to assign all molecular parameters for the cMD using the charmm36-mar2019 force field, while GROMACS 2019.3 was used for further analysis (Department of Biophysical Chemistry, Groningen University, Netherlands) [25]. The structure was put in a triclinic box, adding TIP3P water molecules, and then the system was neutralized with counter ions. The energy minimization was performed in 5000 steps using the steepest descent algorithm (forces less than 100 kJ/mol/nm) [26]. The integration of the MD was at each time step of 2 fs; a V-rescale thermostat and the Nose–Hoover barostat kept the temperature and pressure at 300 K and 1 atm, respectively. The MD run lasted 100 ns to relax the system.

With the “gmx cluster” function implemented in GROMACS 2019.3, the frame most representative of the MD run of the wild-type SAA1was extracted, and it was used as input for the docking simulation with HGA. The SAA-ΔR1 mutant (Arg-1 is not considered as the signal peptide) was obtained using an AI algorithm named AlphaFold (Google DeepMind and Isomorphic Labs, Mountain View, CA, USA; London, UK) [27], providing as input the primary structure of SAA1 without Arg-1. The 3D structure generated by AlphaFold was further optimized as described above.

The 3D structure of HGA was obtained from the PubChem database [28], with PubChem CID 780, and downloaded in sdf format and converted to pdbqt format using the openbabel tool 2.0.2 (Open Babel Team) [29,30]. Currently, no binding molecular information of small molecules on SAA1 is available; thus, to predict the potential binding pose of the HGA on SAA1, we applied a blind docking approach. A box able to enclose the whole SAA 3D structure was created, setting the grid box to the centre of mass of the target using DockingPie2.0 (Department of Biochemical Sciences, Sapienza University, Rome, Italy) [31,32] implemented in PyMOL 3.0 (DeLano Scientific LLC, Schrödinger LLC, New York, NY, USA) using the docking tool Autodock/Vina (Molecular Graphics Lab, Scripps Research Institute, La Jolla, CA, USA) [33,34]. A box able to enclose the whole SAA 3D structure was generated with a dimension of 45 Å (X), 36 Å (Y), and 27 Å (Z), setting the grid to the centre of mass of the target. To obtain a more consistent result from our docking simulation, we changed the default exhaustiveness from 8 to 32, selecting the first 100 best binding poses. All other parameters were used as default. All the potential SAA1/HGA complexes obtained from the docking simulations were saved in PDB format and prepared for the cMD as previously described. Thus, apo-state SAA1, the ΔR1-SAA mutant, and all SAA1/HGA complexes were subjected to a cMD run of 1 µs (1000 ns). All MD analyses were explored with GROMACS 2019.3 packages (Department of Biophysical Chemistry, Groningen University, Groningen, The Netherlands). The Bio3D library implemented in R studio (Posit PBC, Wien, Austria) analyzed the trajectory to provide the system conformational distribution and the principal component analysis (PCA). GRACE generated the MD graphs and PyMOL 3.0 was used as a molecular graphic interface and produced the biological system pictures. PLIP interaction analyzed the interaction network obtained following the docking simulation [35,36].

## 3. Results

### 3.1. HGA Treatment Potentially Induced SAA and SAP Colocalization

To investigate the cellular localization of the proteins SAA and SAP, immunofluorescence staining was conducted on C20/A4 human chondrocytes treated with HGA at various concentrations (0.066 mM and 0.1 mM) for one and two weeks. The concentrations used were selected to mimic the HGA serum levels reported in AKU patients [37] and are consistent with those used in previous AKU cellular models [38,39,40,41,42].

**Figure 1:**
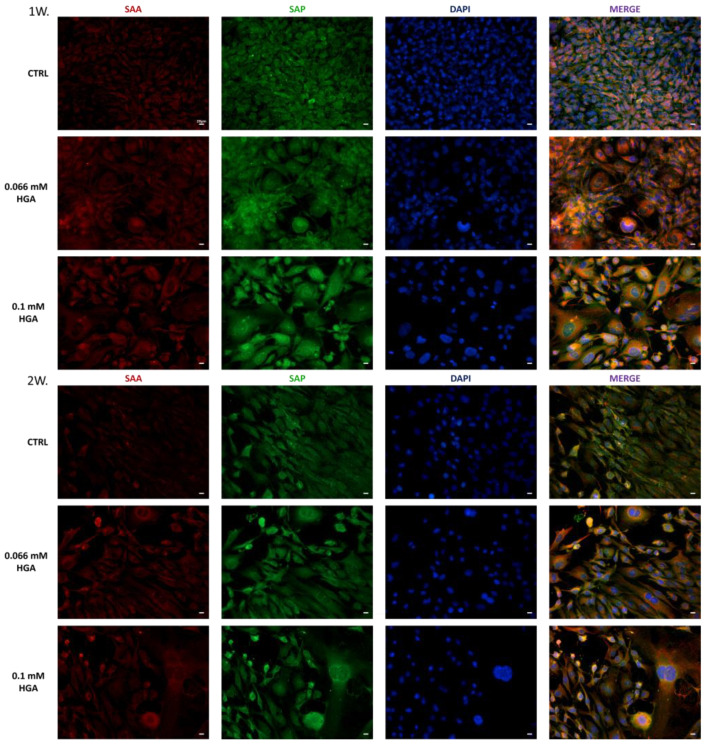
SAA (red) and SAP markers (green) are both double-labeled. SAA and SAP staining were visible in both control and 0.066 and 0.1 mM of HGA-treated cells. After one (1W) and two (2W) weeks of HGA treatment, C20/A4 cells began to lose their organization and exhibit abnormalities in nuclear shapes and morphology. Scale bar = 20 μm.

The red (SAA) and green (SAP) staining were evident in both control and HGA-treated C20/A4 cells, starting from the first week of treatment. In untreated C20/A4 cells, the two proteins appeared to be distributed homogeneously. However, in cells treated with HGA at a concentration of 0.066 mM, and more notably of 0.1 mM, both SAA and SAP seemed to be concentrated primarily in the perinuclear zone of the cells (Figure 1). Moreover, after 1 week of treatment with 0.1 mM of HGA, and more notably after 2 weeks of treatment with 0.066 mM and 0.1 mM of HGA, C20/A4 cells lost their regular shape, becoming enlarged and multinuclear. Additionally, the cell nuclei became irregular and began to lose their well-defined boundaries. Previous studies have shown that HGA induces oxidative stress, which over time reduces the protective autophagic processes in cartilage, ultimately leading to chondroptosis, a specific form of apoptosis, in AKU chondrocytes, resulting in morphological changes [43]. In a prior study, DAPI staining revealed apoptotic nuclei in C20/A4 cells after HGA treatment. Similarly, in this study, nuclear shape alterations were most evident after one and two weeks of HGA treatment. These findings confirmed that HGA acts as an inducer of apoptosis [40].

**Figure 2:**
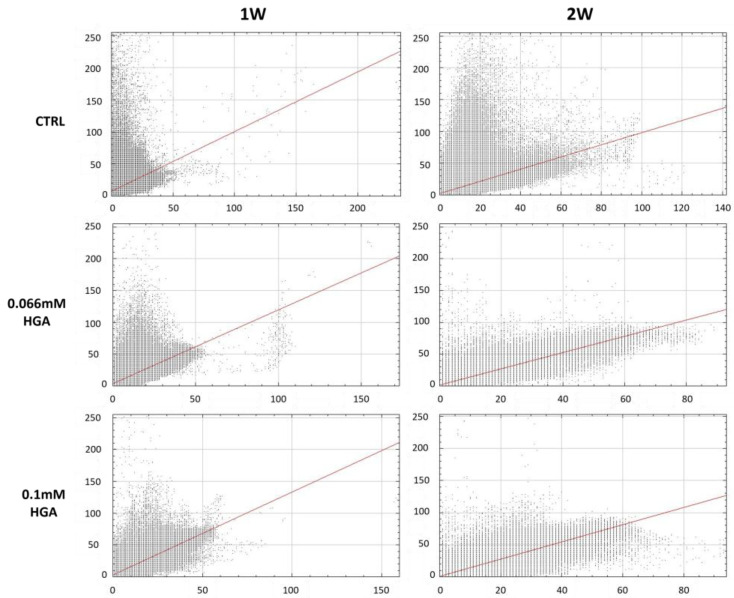
Scatterplot of red (SAA) and green (SAP) pixel intensities of C20/A4 cells following incubation for one (1W) and two (2W) weeks with HGA of 0.066 and 0.1 mM concentrations. As previously stated, the plots of the two proteins indicated a rise in correlation between them, as seen by an increase in linearity that increases with treatment exposure duration and HGA concentration.

Subsequently, a scatterplot analysis was conducted (Figure 2) to determine the correlation between the red (SAA, x-axis) and green (SAP, y-axis) channels of the immunofluorescence data. A scatterplot is a pixel distribution diagram used to analyze the spatial and functional interactions among several molecular markers within cells. Colocalization is demonstrated by the presence of data points that cluster together in a diagonal line, indicating that the two markers are present in the same areas [44]. The analysis revealed that the correlation between the two channels, which shows the level of overlap and hence colocalization between the two proteins, became more linear with increasing HGA concentration and extended treatment duration. This suggested a possible interaction between the two proteins due to HGA treatment.

**Figure 3:**
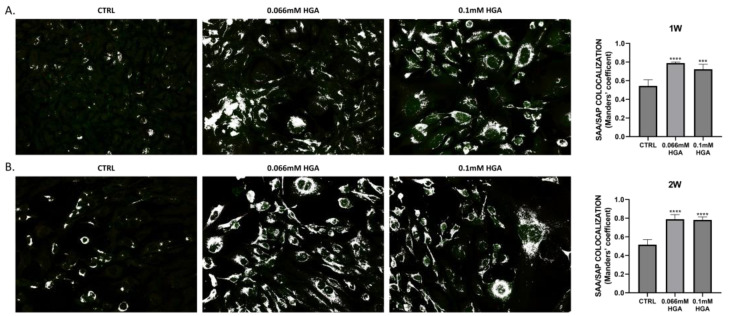
Immunofluorescence analysis was conducted on C20/A4 cells to examine white dot colocalization, employing anti-SAA and anti-SAP antibodies at (**A**) 1 and (**B**) 2 weeks of treatment. Quantitative assessment of SAP colocalization with SAA is depicted in a bar graph utilizing Manders’ coefficient (mean ± standard deviation). Statistically significant differences are shown using *** *p* = 0.0002, **** *p* < 0.0001. Data are displayed as mean ± SD. *p*-values were calculated using ordinary one-way ANOVA with Dunnett’s post hoc test.

White dot colocalization images of SAA and SAP revealed a significant overlap of the two signals (Figure 3). The colocalization was particularly noticeable after two weeks of treatment with HGA, confirming the previously hypothesized interaction between the two proteins. In contrast, untreated cells showed a very low overlap of the two signals despite the presence of both SAA and SAP, as demonstrated in the immunofluorescence analysis.

**Figure 4:**
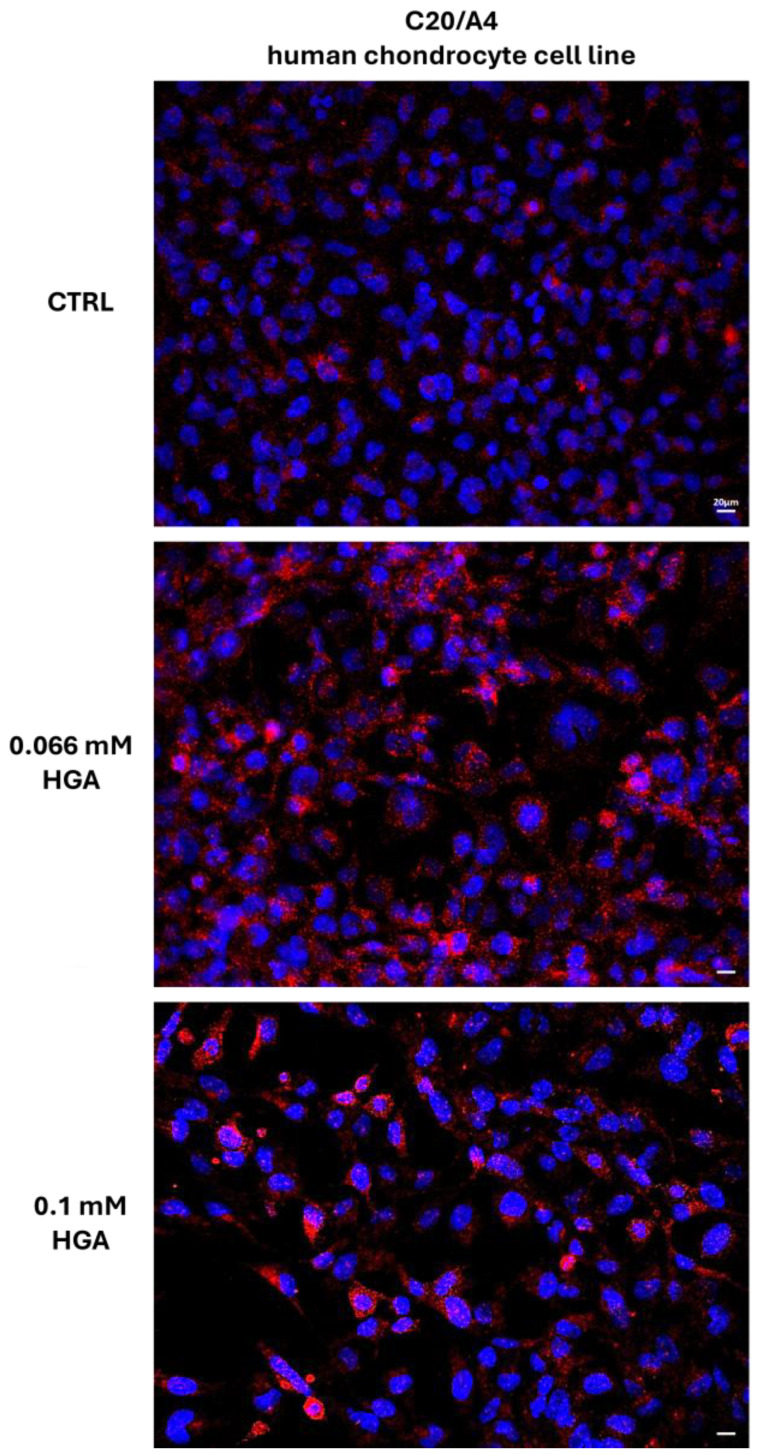
Duolink PLA assay shows colocalization of SAA and SAP. Nuclei are stained with DAPI. Scale bar = 20 μm.

Finally, to demonstrate the close proximity of SAA and SAP, the PLA method was performed on untreated and HGA-treated C20/A4 human chondrocytes (Figure 4). The in situ PLA enables the detection of protein–protein interactions within cells and tissues [45]. This assay reveals colocalization between two proteins separated by less than 30 nm. The strong reaction products, visible as red fluorescent spots, confirmed the colocalization of SAA and SAP. Minimal colocalization was evident throughout the cells in the control group, as indicated by white dot colocalization. In cells treated with 0.066 mM of HGA, colocalization was more noticeable, especially around the nuclei. In cells treated with 0.1 mM of HGA, the red fluorescent spots were predominantly concentrated around the nuclei.

### 3.2. HGA Treatment Promoted Amyloidogenic Protein Aggregation

The presence of SAA and SAP in AKU has been determined, allowing the condition to be classified as a secondary (AA) amyloidosis [6]. SAP is known to bind and stabilize formed amyloid fibrils [46,47]. Immunofluorescence and image analysis suggested not only a change in the cellular spatial arrangement of SAA and SAP, but also a potential interaction between the two proteins, which became more evident with increasing HGA concentration and time exposure.

**Figure 5:**
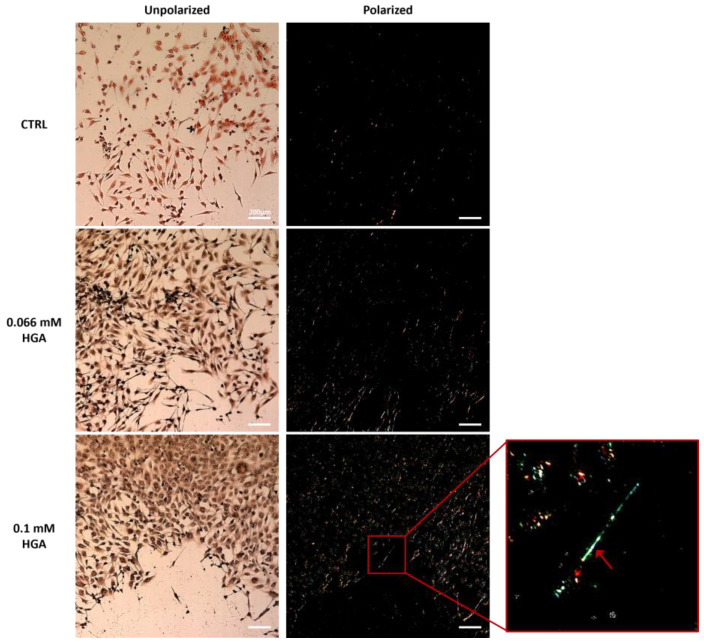
Congo red staining of untreated (CTRL) and HGA-treated C20/A4 cells (0.066 mM and 0.1 mM). In the zoomed-in area, green-yellow staining (arrow) highlights amyloid buildup following HGA treatment. Scale bar = 200 μm.

Congo red staining under polarized light showed green-yellow birefringence in C20/A4 cells treated with 0.066 mM of HGA, and more noticeably with 0.1 mM of HGA after two weeks of treatment, while untreated cells did not display birefringence (Figure 5). This observation might indicate that in HGA-treated C20/A4 cells, SAA and SAP interact and initiate the formation of amyloid fibrils, whereas in untreated cells, the interaction between the two proteins is minimal.

### 3.3. HGA Treatment Triggered SAA Aggregation

Secondary amyloidosis is characterized by the production of amyloid fibrils resulting from SAA monomer binding. It is also known that, in AKU, HGA is a potential aggregation enhancer that promotes intermolecular associations [48].

**Figure 6:**
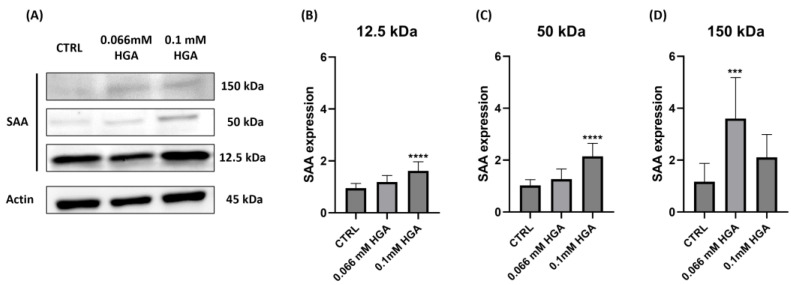
(**A**) SAA expression levels in C20/A4 cells untreated (CTRL) and treated with 0.066 mM and 0.1 mM of HGA for two weeks. (**B**) SAA expression at 12.5 kDa, (**C**) SAA expression at 50 kDa, (**D**) SAA expression at 150 kDa. Data are presented as fold change. Statistically significant differences are indicated by *** *p* = 0.0002 and **** *p* < 0.0001. All experiments were performed in triplicate. *p*-values were calculated using ordinary one-way ANOVA with Dunnett’s post hoc test.

To evaluate the aggregation of SAA in our cellular model, we treated C20/A4 cells with HGA at various concentrations (0.066 mM and 0.1 mM) for two weeks. The expression levels of SAA were determined using a Western blot, considering the monomer (12.5 kDa), tetramer (50 kDa), and high molecular weight (HMW) aggregates (150 kDa). The analysis of the immunoreactive bands revealed that the SAA monomer was present in both untreated and HGA-treated C20/A4 cells, confirming the results observed with immunofluorescence. In particular, cells treated with 0.1 mM of HGA showed significantly higher levels of SAA than the control, indicating that HGA might act as a promoter in the synthesis of SAA (Figure 6B). Moreover, treatment with 0.1 mM of HGA increased the expression of the SAA tetramer by 2-fold compared to the control (Figure 6C). On the contrary, the expression of HMW aggregates was significantly higher in cells treated with 0.066 mM of HGA (4-fold increase).

### 3.4. In Silico Results

#### 3.4.1. Structural Resources and Docking Simulation

The 3D structure of human serum amyloid A1 obtained with the X-ray diffraction method and a resolution of 2.19 Å was downloaded from the RSCB PDB database (PDB code: 4IP8). The 3D structure was optimized through molecular modeling to add and/or adjust potential missing side chains; thus, an energy minimization was performed to lead the system to the minimum energy. The SAA1-∆R1 mutant 3D structure was generated and optimized as described for wild-type SAA1 prior to removing the R1 residue from the primary structure.

**Figure 7:**
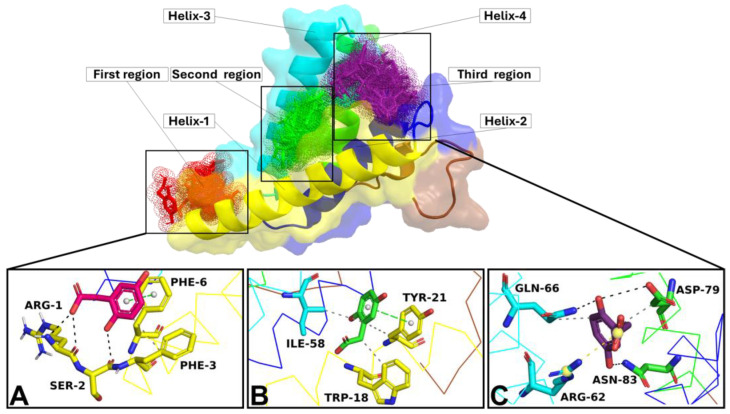
SAA1/HGA blind docking overview. The SAA1 is reported in yellow (helix 1), blue (helix 2), cyan (helix 3), green (helix 4), and brown (C-ter) image/surface. The red (first region), green (second region), and purple (third region) dots represent the binding regions identified by the blind docking simulation; all binding poses obtained following the docking simulation are shown inside of the corresponding dots. The best binding pose of HGA in the (**A**) first region, (**B**) second region, and (**C**) third region is reported in red, green, and purple sticks, respectively. The binding residues are shown in sticks and the carbon atom is the same colour as the helix it belongs to, while the oxygen, nitrogen, and polar hydrogen atoms are coloured in red, blue, and white, respectively. The hydrophobic interaction, h-bonds, π-stacking, and salt bridge are shown as grey, black, green, and yellow dotted lines.

The human SAA1 3D structure was used as the starting structure to obtain the SAA1/HGA complex. To identify the potential binding site of HGA on the target, a blind docking approach was firstly used. Thus, HGA was able to dock the overall SAA1 surface. Docking results provided the ability of HGA to predominantly dock in three different SAA1 regions: on the N-terminal region (helix 1) (first region), between helices 2 and 3 (second region), and between helices 2, 3, and 4 (third region) (Figure 7). A rational docking was performed using the three best binding poses docked in the corresponding regions as reference to generate a box able to enclose these SAA1 binding regions. The best binding poses obtained from the docking simulation showed that HGA was able to trigger a hydrophobic and polar interaction network within each binding region exhibiting an affinity of −3.9 kcal/mol (first region) (Figure 7A), −4.4 kcal/mol (second region) (Figure 7B), and −5.6 kcal/mol (third region) (Figure 7C); thus, the three complexes were considered for further in silico analyses.

#### 3.4.2. cMD: Structural Stability and HGA Binding Pose Stability

To dissect molecular insights regarding the SAA1 structural conformations and the SAA1/HGA interaction, a cMD run of 1 µs was performed for each biological system (wild-type SAA1, the SAA1-∆R1 mutant, and the three SAA1/HGA complexes). To prevent potential computational bias, the structural integrity of the target backbone for each system and the stability of the HGA binding pose was computed through distance, RMSD, and intramolecular h-bond analyses. To evaluate the binding pose stability of HGA docked in the three different regions, an analysis considering the distance average between the backbone and the HGA center of mass along the MD run was performed. The distance results showed that HGA docked in the first and second region, leaving the binding region in the first frame of the MD run (interrupted, as it is not useful for the study), while in the third region, HGA showed a good binding stability for 500 ns, then HGA bound in a close region to leave the target after around 800 ns (Figure S1). Thus, the complex of HGA docked in the third SAA1 region was considered for further analyses.

**Figure 8:**
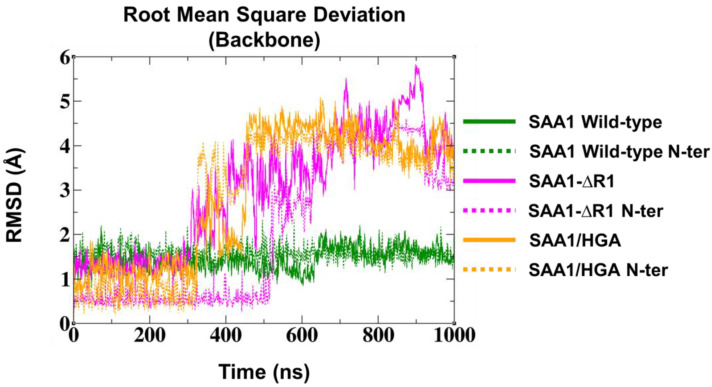
RMSD trend for wild-type SAA1 (green), SAA1-∆R1 mutant (magenta), and SAA1/HGA complex (orange).

RMSD analyses showed a high structural stability for wild-type SAA1, exhibiting a stable trend of 1.5 Å along the entire MD run. Contrarily, the SAA1-∆R1 mutant and the SAA1/HGA complex showed a stable trend for the first 300 ns, then the RMSD values rapidly increased to 4.5 Å until the end of the MD run (Figure 8). To further explore structural insights, the stability of the N-ter region was examined. From the RMSD results, the wild-type SAA1 N-ter region was highly stable with a RMSD value of 1.5 Å during the MD run. Contrarily, the SAA-∆R1 mutant and the SAA1/HGA complex showed a stable trend for the first 520 ns and 320 ns, respectively, then the RMSD values increased quickly, achieving values of 4 Å for both the systems (Figure 8).

The intramolecular h-bond interaction network of the N-ter region confirmed its high structural instability for the SAA1-∆R1 mutant and the SAA1/HGA complex N-ter region, showing for both a decrease in the h-bond number from nine to zero, while the SAA wild type exhibited a constant number of h-bonds along the entire MD run (Figure S2).

#### 3.4.3. Principal Component (PC), Conformational, and H-bond Network Analyses

**Figure 9:**
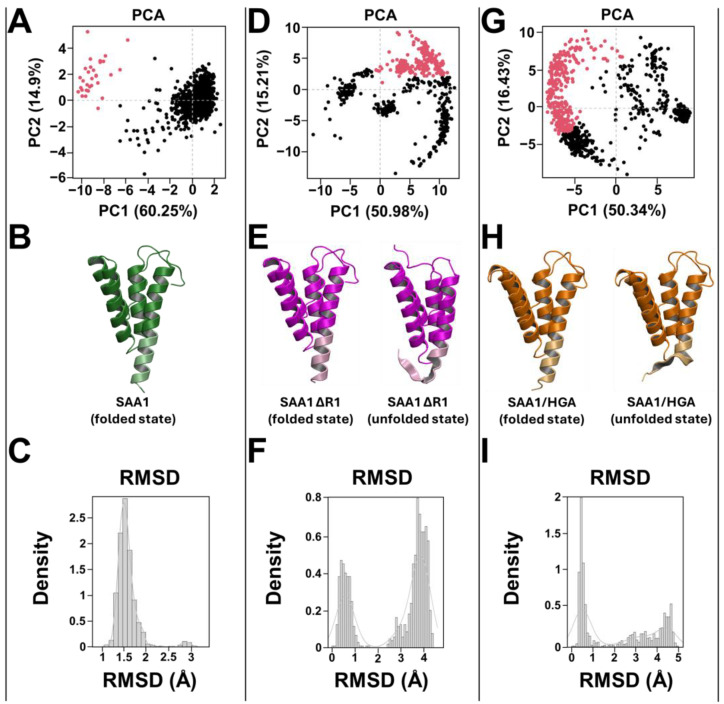
Principal component, conformational, and RMSD distribution analyses. PCAs of (**A**) SAA1 wild type, (**D**) SAA1-∆R1 mutant, and (**G**) SAA1/HGA complex. The black and red points represent the PC1 and PC2, respectively. Conformational analysis of (**B**) SAA1 wild type (green image), (**E**) SAA1-∆R1 mutant (magenta image), and (**H**) SAA1/HGA complex (orange image). The N-ter region of each system was reported with a color lighter. Only SAA1-∆R1 mutant and SAA1/HGA complex had a N-ter unfolded state. The conformational distribution based on the RMSD values was reported for (**C**) SAA1 wild type, (**F**) SAA1-∆R1 mutant, and (**I**) SAA1/HGA complex.

To further investigate the N-ter region features of the systems, PCA studies were performed. PCAs of MD trajectories of each system considering the N-ter region revealed that the eigenvectors of each structure possessed significant values (Figure S3A–C). In particular, the first three eigenvectors represented 85.6% (wild-type SAA1) (Figure S3A), 77.2% (SAA1-∆R1 mutant) (Figure S3B), and 76.8% (SAA1/HGA complex) (Figure S3C) of the entire conformational population. Therefore, most of the conformations of wild-type SAA1 were confined within a subspace of very small dimensions (Figure 9A), which differs for the SAA1-∆R1 mutant and the SAA1/HGA complex, which showed two different subspaces (Figure 9D,G). RMSD distribution analyses, performed for each system, confirmed PCAs, showing a clear presence of one only conformational population (folded state) for SAA1 wild-type (Figure 9B-C) and two main conformational states (folded and unfolded states) for the SAA1-∆R1 mutant (Figure 9E,F) and the SAA1/HGA complex (Figure 9H,I).

Conformational analyses were also conducted by using the PyMOL 3.0 3D molecular graphical interface software analyzing all MD trajectories. Wild-type SAA1 showed a high structural stability along the MD run, exhibiting one only conformation, and no misfolding process was observed for the N-ter region, confirming PCA analyses (Video S1). Contrarily, the SAA1-∆R1 mutant and SAA1/HGA complex revealed two different conformations along the MD run; in detail, the SAA1-∆R1 mutant was stable for the first 530 ns, then the N-ter region (from Ser-1 to Phe-11) started its misfolding process to completely lose its secondary structure around 637 ns (Video S2). While the SAA1/HGA complex was stable for the first 325 ns, its misfolding started and fully lost its N-terminal 3D structure (from Arg-1 to Phe-11) around 470 ns (Video S3).

**Figure 10:**
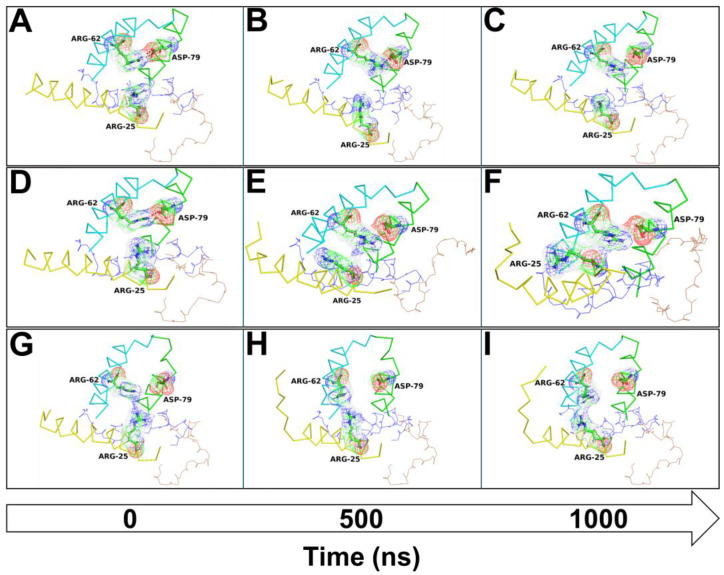
Arg-25, Arg-62, and Asp-79 contact overview along the MD run. The SAA1 is reported in yellow (helix 1), blue (helix 2), cyan (helix 3), green (helix 4), and brown (C-ter) ribbon/lines. The Arg-25, Arg-62, and Asp-79 are shown as a green (carbon atom), red (oxygen atom), blue (nitrogen atom), or white (hydrogen atom), stick/mesh. The SAA1 wild type, SAA1-∆R1 mutant, and SAA1/HGA complex residue contact/interactions with the initial frame (**A**,**D**,**G**), middle MD run (**B**,**E**,**H**), and last frame (**C**,**F**,**I**), are reported along the entire MD run. The arrow indicates the timeline of the MD run.

The molecular basis of the N-ter misfolding process of the SAA1-∆R1 mutant and the SAA1/HGA complex were further analyzed evaluating the intramolecular contact and hydrogen bond interaction using the function “gmx hbond” implemented in GROMCAS 2019.3. H-bond analyses provided evidence of a different intramolecular interaction network for wild-type SAA1 compared with other systems. It showed a strong and stable electrostatic h-bond interaction network between the Arg-62 (helix 3) and Asp-79 (helix 4) residues along the entire MD run (Figure 10A–C and Video S4); contrarily, the SAA1-∆R1 mutant exhibited only a weak electrostatic interaction between Arg-62 and Asp-79 (Figure 10D–F and Video S5), while the SAA1/HGA complex did not show any interaction between Arg-62 and Asp-79 along the entire MD run (Figure 10G–I and Video S6). Interestingly, a correlation between the Arg-62 and Asp-79 interaction was detected with Arg-25, located on the N-ter helix, which would seem to be involved in the formation of the Arg-62/Asp-79 interaction upon formation of an electrostatic contact with Arg-62, influencing the N-ter region stability.

In fact, in wild-type SAA1, Arg-25 and Arg-62 never showed any contact, the SAA1-∆R1 mutant presented an Arg-25/Arg-62 contact for a moderate time, while the SAA1/HGA complex exhibited an Arg-25/Arg-62 interaction for the entire MD run, orienting the Arg-62 side chain toward helix 1 (N-ter helix) (Figure 10A–I and Videos S4–S6).

## 4. Discussion

AKU is a rare autosomal recessive degenerative disease that significantly affects several organ systems, particularly the osteoarticular system, with cartilage being notably more susceptible to damage compared to bone [49]. Ochronosis, an essential feature of AKU, includes a cascade of effects involving the weakening of cartilage, increased stiffness, increased susceptibility to fractures, chronic inflammation, and ultimately, the emergence of osteoarthritis [50]. Chondrocytes, cartilage’s main cells, play an important role in coordinating the synthesis of extracellular matrix components such as collagen and proteoglycan. These components are critical for cartilage’s structural integrity and biomechanical resiliency, which is required to tolerate mechanical stress. Nonetheless, performing research on human chondrocytes has various challenges, most notably the difficulty in collecting cells from AKU patients’ biopsies. The issues are complicated by variability among donors, which can be related to factors like age and pre-existing medical disorders. The scarcity of AKU cases, procuring biological specimens, and other factors make AKU research highly challenging in controlled laboratory settings, limiting our capacity to compare and apply research findings to a larger population. AKU-related events have been investigated by evaluating cells directly collected from AKU patients’ biopsies or by utilizing cellular models that recreate the disease scenario. The adoption of an immortalized cell line of human chondrocytes as a cellular model for AKU has created new perspectives for exploring this rare condition [40,51], reducing the necessity for biopsy-derived samples.

In recent years, the AKU/amyloidosis relationship has been proposed [7], as well as the evidence of the presence of SAA and SAP in AKU, which allowed for the classification of AKU as a secondary (AA) amyloidosis. Amyloidosis is a pathological progression caused by the conversion of normally soluble proteins into insoluble protein fibrillar aggregates, named amyloids, that deposit either systemically or in a specific organ and may be associated with several disorders.

The purpose of this study was to further investigate the AKU-related amyloidosis mechanisms and the development of SAA aggregates and their impact on the formation of amyloid fibrils. Furthermore, the role of HGA in amyloidogenic processes [52,53] was also investigated.

Here, we suggest new evidence of HGA amyloidogenic action, confirming the metabolite’s preclinical significance in the development of AKU-associated SAA amyloidosis. In addition to modifications caused by HGA, such as changes in cell organization, anomalies in nuclear borders, and cell morphology [40,42], our findings from immunofluorescence demonstrated that SAA and SAP were produced in control and treated cells. The two proteins were uniformly distributed in human C20/A4 control chondrocytes; previous studies focused on the intrinsic generation of SAA by these cells, caused by their cancerous features [54,55,56,57]. Nevertheless, when the cells were exposed to HGA, SAA and SAP become mostly concentrated in certain areas, particularly around the nucleus, as has already been observed in AKU chondrocytes [42], confirming the role of HGA in the aggregation of SAA and also indicating a possible physical connection between SAA and SAP. Similarly, we noted a comparable pattern for SAP. The relationship between the two proteins was further strengthened by the correlation analysis, where the scatterplot (JaCoP plugin, ImageJ software) revealed an increase in linearity between the red and green channels (SAA and SAP, respectively). This observation was substantiated with an analysis using ImageJ software (colocalization plugin), where white dot colocalization indicated a heightened overlap between the two channels, and the Manders’ coefficient for SAP overlapping with SAA approached in HGA-treated cells was compared to untreated ones. These findings suggest a potential interaction between the two proteins, an interaction seemingly induced by HGA treatment, as evidenced by the persistence of lower white dot colocalization and Manders’ coefficient values in untreated cells at both 1- and 2-week intervals. The Duolink PLA analysis confirmed the results observed with immunofluorescence and the ImageJ analysis. In untreated human C20/A4 chondrocytes, minimal and homogeneously distributed colocalization was observed, indicated by red fluorescent spots representing a distance of less than 30 nm between the two proteins. Notably, cells treated with 0.066 mM, and especially 0.1 mM of HGA, showed an increase in colocalization, particularly in the area surrounding the nucleus. This suggests a potential role of HGA as an enhancer of SAA and SAP interaction and amyloid fibril formation. These results indicate that HGA may have an essential role in AKU amyloidosis by binding to amyloid proteins and facilitating the onset of amyloid fibrils.

Our results were supported by Congo red staining, which confirmed the presence of amyloid fibril structures only in HGA-treated cells, as evidenced by green/yellow staining, whereas similar structures were absent in controls. Our theory states that while SAA and SAP proteins are generated in control cells, their interaction, which eventually leads to amyloid formation, does not seem to occur in the absence of HGA-induced effects. This is substantiated by the lack of Congo red staining under polarized light, indicating the inability to assist aggregation and subsequent amyloid fibril production in the absence of HGA. Our outcomes are consistent with previous studies on the same proteins subjected to various aggregating events [58].

A WB analysis after 14 days of HGA treatment revealed minimal aggregation under control conditions. In contrast, cells treated with 0.066 mM and 0.1 mM of HGA showed increased levels of the SAA monomer, oligomers, and HMW, thus leading to the potential generation of amyloid fibrils. The results were consistent with previous in vitro observations [48], which revealed that HGA accelerated the rate of amyloid fibril formation in a time- and dose-dependent manner and that the fibrillization of amyloidogenic proteins and peptides incubated with HGA was eventually faster than that of their untreated counterparts.

In previous work, in vitro methods showed that amylogenic SAA1 (SAA variant lacking an arginine at the N-terminus) was characterized by modifications to the N-terminal region, which greatly influenced the pathogenesis of AA amyloidosis [16]. Thus, alterations of the SAA1 N-ter region influence its amyloidogenic properties.

In this study, we coupled docking with cMD to identify the potential N-ter region misfolding of SAA1 and its interaction with HGA. An analysis of the crystal structure of the SAA1 reveals that the protein has a relatively flat surface, which would be unsuitable for HGA binding. A blind docking simulation identified three possible binding regions for HGA, and cMD investigated their suitability. MD simulations disproved the HGA binding pose in the first and second region of the target, as HGA left the binding region in the first frame of the MD, while a good HGA binding pose stability was revealed in the third region.

The comparison of the backbone structural stability of the three systems considered in this study showed high stability for wild-type SAA1; conversely, the SAA-∆R1 mutant and the SAA1/HGA complex resulted in being unstable structurally due to the misfolding of the N-ter region. Interestingly, the SAA-∆R1 mutant and the SAA1/HGA complex showed a similar misfolding of the N-ter region; however, the SAA1/HGA complex exhibited a misfolding process faster than the SAA1-∆R1 mutant.

The SAA1 N-ter region misfolding is currently unclear; however, in this study, some molecular insights would seem to elucidate this process.

In the wild-type SAA1, Arg-1 did not show significant interactions with neighboring residues, but a strong tendency to point inward of the N-ter helix, characterized by numerous residues having net negative/positive charges (Arg-1, Glu-9, Asp-12, Arg-15, Asp-16, Arg-19, Asp-23, Arg-25, and Glu-26).

The SAA-∆R1 mutant showed the N-ter region misfolding after 520 ns of the cMD run, likely caused by an incorrect electrostatic distribution due to the lack of the net positive charge of Arg-1, as proposed in the previous work [16].

Meanwhile, the SAA1/HGA complex showed the N-ter region misfolding after 320 ns of the cMD run, exhibiting a tendency to misfold faster than the SAA-∆R1 mutant. Such a tendency could be explained by the different starting conformation of the SAA1, caused by the HGA binding. HGA binding between helix 3 and helix 4 causes a different orientation of the residue side chains, disallowing a strong and stable electrostatic and h-bond interaction network between the Arg-62 (helix 3) and Asp-79 (helix 4) residues established in wild-type SAA1. In particular, the lack of the Arg-62/Asp-79 interaction would orient the Arg-62 side-chain toward helix 1 (N-ter region), likely causing an electrostatic distribution alteration like in the SAA-∆R1 mutant, leading to the N-ter region misfolding. Thus, HGA would quicken the misfolding process by orienting Arg-62 toward the N-ter region, behaving as an enhancer of the fibril formation/aggregation process and the amyloidosis progression, confirming in vitro evidence.

## 5. Conclusions 

In the present study, applying comprehensive in vitro and in silico approaches, we provided new details about the potential role of HGA as an aggregation enhancer for the amyloidogenic process. The HGA-dependent modifications observed during amyloid formation indicated the ability of HGA to promote fibril formation/aggregation upon binding with SAA1 and its N-ter region misfolding, as suggested from in silico studies. Finally, our findings suggest that HGA contributed to the effectiveness of aggregation events that characterize secondary amyloidosis in AKU, as well as a possible explanation for the clinically confirmed beginning of amyloidosis in AKU patients. Our thorough investigation has revealed the links between HGA-induced ochronosis and amyloidogenic processes associated with AKU, offering important insights into the pathophysiological mechanisms underlying AKU progression. The use of various analytical approaches has increased our understanding of AKU pathophysiology, emphasizing the important role of cellular models in comprehending the complexities of rare genetic diseases.

## Data Availability

No new data were created or analyzed in this study. Data sharing is “not applicable” to this article.

## Supplementary Materials

The following supporting information can be downloaded at https://www.mdpi.com/article/10.3390/cells13171501/s1, Figure S1. Distance average between the Backbone and HGA centre of mass along the MD run. Figure S2. Intramolecular hydrogen bond network of the SAA1 N-ter region along the MD run. Figure S3. Eigenvalues rank. (A) wild type SAA1, (B) SAA1-∆R1 mutant, and (C) SAA1/HGA complex. Video S1. SAA1 wild type MD run. SAA1 wild type was reported as a green cartoon, while the N-ter region was shown in light green. Video S2. SAA1 ∆R1 mutant MD run. SAA1 ∆R1 mutant was reported as a magenta cartoon, while the N-ter region was shown in pink. Video S3. SAA1/HGA complex MD run. SAA1/HGA complex was reported as an orange cartoon, while the N-ter region was shown in light orange. HGA was represented as an orange line and dots. Video S4. SAA1 wild type Arg-25, Arg-62, and Asp-79 contact overview along the MD run. The SAA1 was reported in yellow (helix 1), blue (helix 2), cyan (helix 3), green (helix 4), and brown (C-ter) ribbon/lines. The Arg-25, Arg-62, and Asp-79 were shown as a yellow/cyan/green (carbon atom), red (oxygen atom), blue (nitrogen atom), white (hydrogen atom), stick/mesh. Video S5. SAA1/HGA complex Arg-25, Arg-62, and Asp-79 contact overview along the MD run. The SAA1 was reported in yellow (helix 1), blue (helix 2), cyan (helix 3), green (helix 4), and brown (C-ter) ribbon/lines. The Arg-25, Arg-62, and Asp-79 were shown as a yellow/cyan/green (carbon atom), red (oxygen atom), blue (nitrogen atom), white (hydrogen atom), stick/mesh. Video S6. SAA1 ∆R1 mutant Arg-25, Arg-62, and Asp-79 contact overview along the MD run. The SAA1 was reported in yellow (helix 1), blue (helix 2), cyan (helix 3), green (helix 4), and brown (C-ter) ribbon/lines. The Arg-25, Arg-62, and Asp-79 were shown as a yellow/cyan/green (carbon atom), red (oxygen atom), blue (nitrogen atom), white (hydrogen atom), stick/mesh.

## References

[1] Bernardini G., Braconi D., Zatkova A., Sireau N., Kujawa M.J., Introne W.J., Spiga O., Geminiani M., Gallagher J.A., Ranganath L.R. (2024). Alkaptonuria. Nat. Rev. Dis. Primers.

[2] Milella M.S., Geminiani M., Trezza A., Visibelli A., Braconi D., Santucci A. (2024). Alkaptonuria: From Molecular Insights to a Dedicated Digital Platform. Cells.

[3] Spiga O., Cicaloni V., Zatkova A., Millucci L., Bernardini G., Bernini A., Marzocchi B., Bianchini M., Zugarini A., Rossi A. (2018). A new integrated and interactive tool applicable to inborn errors of metabolism: Application to alkaptonuria. Comput. Biol. Med..

[4] Mistry J.B., Bukhari M., Taylor A.M. (2013). Alkaptonuria. Rare Dis..

[5] Bernini A., Petricci E., Atrei A., Baratto M.C., Manetti F., Santucci A. (2021). A molecular spectroscopy approach for the investigation of early phase ochronotic pigment development in Alkaptonuria. Sci. Rep..

[6] Millucci L., Spreafico A., Tinti L., Braconi D., Ghezzi L., Paccagnini E., Bernardini G., Amato L., Laschi M., Selvi E. (2012). Alkaptonuria is a novel human secondary amyloidogenic disease. Biochim. Biophys. Acta..

[7] Millucci L., Braconi D., Bernardini G., Lupetti P., Rovensky J., Ranganath L., Santucci A. (2015). Amyloidosis in alkaptonuria. J. Inherit. Metab. Dis..

[8] Webb N.R. (2021). High-Density Lipoproteins and Serum Amyloid A (SAA). Curr. Atheroscler. Rep..

[9] Blancas-Mejía L.M., Ramirez-Alvarado M. (2013). Systemic amyloidoses. Annu. Rev. Biochem..

[10] Scarpioni R., Ricardi M., Albertazzi V. (2016). Secondary amyloidosis in autoinflammatory diseases and the role of inflammation in renal damage. World J. Nephrol..

[11] Real de Asúa D., Costa R., Galván J.M., Filigheddu M.T., Trujillo D., Cadiñanos J. (2014). Systemic AA amyloidosis: Epidemiology, diagnosis, and management. Clin. Epidemiol..

[12] Simons J.P., Al-Shawi R., Ellmerich S., Speck I., Aslam S., Hutchinson W.L., Mangione P.P., Disterer P., Gilbertson J.A., Hunt T. (2013). Pathogenetic mechanisms of amyloid A amyloidosis. Proc. Natl. Acad. Sci. USA.

[13] Haines M.S., Ramirez E., Moore K.B., Fortin J.S. (2022). Revisiting misfolding propensity of serum amyloid A1, Special focus on the signal peptide region. Biochem. Biophys. Rep..

[14] Sun L., Ye R.D. (2016). Serum amyloid A1, Structure, function and gene polymorphism. Gene.

[15] Sack G.H. (2018). Serum amyloid A—A review. Mol. Med..

[16] Tanaka M., Takarada T., Nadanaka S., Kojima R., Hosoi K., Machiba Y., Kitagawa H., Yamada T. (2023). Influences of amino-terminal modifications on amyloid fibril formation of human serum amyloid A. Arch. Biochem. Biophys..

[17] Tanaka M., Kawakami T., Okino N., Sasaki K., Nakanishi K., Takase H., Yamada T., Mukai T. (2018). Acceleration of amyloid fibril formation by carboxyl-terminal truncation of human serum amyloid A. Arch. Biochem. Biophys..

[18] Röcken C., Menard R., Bühling F., Vöckler S., Raynes J., Stix B., Krüger S., Roessner A., Kähne T. (2005). Proteolysis of serum amyloid A and AA amyloid proteins by cysteine proteases: Cathepsin B generates AA amyloid proteins and cathepsin L may prevent their formation. Ann. Rheum. Dis..

[19] Stix B., Kähne T., Sletten K., Raynes J., Roessner A., Röcken C. (2001). Proteolysis of AA Amyloid Fibril Proteins by Matrix Metalloproteinases-1,-2, and-3. Am. J. Pathol..

[20] UniProt Consortium (2023). UniProt: The Universal Protein Knowledgebase in 2023. Nucleic Acids Res..

[21] Lu J., Yu Y., Zhu I., Cheng Y., Sun P.D. (2014). Structural mechanism of serum amyloid A-mediated inflammatory amyloidosis. Proc. Natl. Acad. Sci. USA.

[22] Janson G., Paiardini A. (2021). PyMod 3, A complete suite for structural bioinformatics in PyMOL. Bioinformatics.

[23] Laskowski R.A., MacArthur M.W., Moss D.S., Thornton J.M. (1993). PROCHECK: A program to check the stereochemical quality of protein structures. J. Appl. Crystallogr..

[24] Jo S., Kim T., Iyer V.G., Im W. (2008). CHARMM-GUI: A web-based graphical user interface for CHARMM. J. Comput. Chem..

[25] Van Der Spoel D., Lindahl E., Hess B., Groenhof G., Mark A.E., Berendsen H.J.C. (2005). GROMACS: Fast, flexible, and free. J. Comput. Chem..

[26] Trezza A., Spiga O., Mugnai P., Saponara S., Sgaragli G., Fusi F. (2022). Functional, electrophysiology, and molecular dynamics analysis of quercetin-induced contraction of rat vascular musculature. Eur. J. Pharmacol..

[27] Jumper J., Evans R., Pritzel A., Green T., Figurnov M., Ronneberger O., Tunyasuvunakool K., Bates R., Žídek A., Potapenko A. (2021). Highly accurate protein structure prediction with AlphaFold. Nature.

[28] Kim S., Chen J., Cheng T., Gindulyte A., He J., He S., Li Q., Shoemaker B.A., Thiessen P.A., Yu B. (2023). PubChem 2023 update. Nucleic Acids Res..

[29] O’Boyle N.M., Banck M., James C.A., Morley C., Vandermeersch T., Hutchison G.R. (2011). Open Babel: An Open chemical toolbox. J. Cheminform..

[30] Fusi F., Durante M., Spiga O., Trezza A., Frosini M., Floriddia E., Teodori E., Dei S., Saponara S. (2016). In vitro and in silico analysis of the vascular effects of asymmetrical N,N-bis(alkanol)amine aryl esters, novel multidrug resistance-reverting agents. Naunyn. Schmiedebergs Arch. Pharmacol..

[31] Rosignoli S., Paiardini A. (2022). DockingPie: A consensus docking plugin for PyMOL. Bioinformatics.

[32] Trezza A., Geminiani M., Cutrera G., Dreassi E., Frusciante L., Lamponi S., Spiga O., Santucci A. (2024). A Drug Discovery Approach to a Reveal Novel Antioxidant Natural Source: The Case of Chestnut Burr Biomass. Int. J. Mol. Sci..

[33] Trott O., Olson A.J. (2010). AutoDock Vina: Improving the speed and accuracy of docking with a new scoring function, efficient optimization and multithreading. J. Comput. Chem..

[34] Carullo G., Ahmed A., Trezza A., Spiga O., Brizzi A., Saponara S., Fusi F., Aiello F. (2021). A multitarget semi-synthetic derivative of the flavonoid morin with improved in vitro vasorelaxant activity: Role of CaV1.2 and KCa1.1 channels. Biochem. Pharmacol..

[35] Salentin S., Schreiber S., Haupt V.J., Adasme M.F., Schroeder M. (2015). PLIP: Fully automated protein–ligand interaction profiler. Nucleic Acids Res..

[36] Fusi F., Trezza A., Spiga O., Sgaragli G., Bova S. (2017). Cav1.2 channel current block by the PKA inhibitor H-89 in rat tail artery myocytes via a PKA-independent mechanism: Electrophysiological, functional, and molecular docking studies. Biochem. Pharmacol..

[37] Ranganath L.R., Milan A.M., Hughes A.T., Dutton J.J., Fitzgerald R., Briggs M.C., Bygott H., Psarelli E.E., Cox T.F., Gallagher J.A. (2016). Suitability Of Nitisinone In Alkaptonuria 1 (SONIA 1): An international, multicentre, randomised, open-label, no-treatment controlled, parallel-group, dose-response study to investigate the effect of once daily nitisinone on 24-h urinary homogentisic acid excretion in patients with alkaptonuria after 4 weeks of treatment. Ann. Rheum. Dis..

[38] Tinti L., Taylor A.M., Santucci A., Wlodarski B., Wilson P.J., Jarvis J.C., Fraser W.D., Davidson J.S., Ranganath L.R., Gallagher J.A. (2011). Development of an in vitro model to investigate joint ochronosis in alkaptonuria. Rheumatology.

[39] Gambassi S., Geminiani M., Thorpe S.D., Bernardini G., Millucci L., Braconi D., Orlandini M., Thompson C.L., Petricci E., Manetti F. (2017). Smoothened-antagonists reverse homogentisic acid-induced alterations of Hedgehog signaling and primary cilium length in alkaptonuria. J. Cell. Physiol..

[40] Galderisi S., Milella M.S., Rossi M., Cicaloni V., Rossi R., Giustarini D., Spiga O., Tinti L., Salvini L., Tinti C. (2022). Homogentisic acid induces autophagy alterations leading to chondroptosis in human chondrocytes: Implications in Alkaptonuria. Arch. Biochem. Biophys..

[41] Galderisi S., Cicaloni V., Milella M.S., Millucci L., Geminiani M., Salvini L., Tinti L., Tinti C., Vieira O.V., Alves L.S. (2021). Homogentisic acid induces cytoskeleton and extracellular matrix alteration in alkaptonuric cartilage. J. Cell. Physiol..

[42] Geminiani M., Gambassi S., Millucci L., Lupetti P., Collodel G., Mazzi L., Frediani B., Braconi D., Marzocchi B., Laschi M. (2017). Cytoskeleton Aberrations in Alkaptonuric Chondrocytes. J. Cell. Physiol..

[43] Millucci L., Giorgetti G., Viti C., Ghezzi L., Gambassi S., Braconi D., Marzocchi B., Paffetti A., Lupetti P., Bernardini G. (2015). Chondroptosis in alkaptonuric cartilage. J. Cell. Physiol..

[44] Bolte S., Cordelières F.P. (2006). A guided tour into subcellular colocalization analysis in light microscopy. J. Microsc..

[45] Söderberg O., Gullberg M., Jarvius M., Ridderstråle K., Leuchowius K.J., Jarvius J., Wester K., Hydbring P., Bahram F., Larsson L.G. (2006). Direct observation of individual endogenous protein complexes in situ by proximity ligation. Nat. Methods.

[46] Tennent G.A., Lovat L.B., Pepys M.B. (1995). Serum amyloid P component prevents proteolysis of the amyloid fibrils of Alzheimer disease and systemic amyloidosis. Med. Sci..

[47] Westermark P., Benson M.D., Buxbaum J.N., Cohen A.S., Frangione B., Ikeda S., Masters C.L., Merlini G., Saraiva M.J., Sipe J.D. (2005). Nomenclature Committee of the International Society of Amyloidosis. Amyloid: Toward terminology clarification. Report from the Nomenclature Committee of the International Society of Amyloidosis. Amyloid.

[48] Braconi D., Millucci L., Bernini A., Spiga O., Lupetti P., Marzocchi B., Niccolai N., Bernardini G., Santucci A. (2017). Homogentisic acid induces aggregation and fibrillation of amyloidogenic proteins. Biochim. Biophys. Acta Gen. Subj..

[49] Fisher A.A., Davis M.W. (2004). Alkaptonuric Ochronosis with Aortic Valve and Joint Replacements and Femoral Fracture: A Case Report and Literature Review. Clin. Med. Res..

[50] Gallagher J.A., Ranganath L.R., Boyde A. (2015). Lessons from rare diseases of cartilage and bone. Curr. Opin. Pharmacol..

[51] Braconi D., Laschi M., Taylor A.M., Bernardini G., Spreafico A., Tinti L., Gallagher J.A., Santucci A. (2010). Proteomic and redox-proteomic evaluation of homogentisic acid and ascorbic acid effects on human articular chondrocytes. J. Cell Biochem..

[52] Millucci L., Ghezzi L., Paccagnini E., Giorgetti G., Viti C., Braconi D., Laschi M., Geminiani M., Soldani P., Lupetti P. (2014). Amyloidosis, inflammation, and oxidative stress in the heart of an alkaptonuric patient. Mediators Inflamm..

[53] Braconi D., Bernardini G., Bianchini C., Laschi M., Millucci L., Amato L., Tinti L., Serchi T., Chellini F., Spreafico A. (2012). Biochemical and proteomic characterization of alkaptonuric chondrocytes. J. Cell Physiol..

[54] Lee J., Beatty G.L. (2021). Serum Amyloid A Proteins and Their Impact on Metastasis and Immune Biology in Cancer. Cancers.

[55] Lin H.Y., Tan G.Q., Liu Y., Lin S.Q. (2019). The prognostic value of serum amyloid A in solid tumors: A meta-analysis. Cancer Cell Int..

[56] Malle E., Sodin-Semrl S., Wcislo-Dziadecka A. (2009). Serum amyloid A: An acute-phase protein involved in tumour pathogenesis. Cell. Mol. Life Sci..

[57] Moshkovskii S.A. (2012). Why do cancer cells produce serum amyloid A acute-phase protein?. Biochemistry.

[58] Wetzel R., Shivaprasad S., Williams A.D. (2007). Plasticity of amyloid fibrils. Biochemistry.

