# Supplementary figures and images for: HGA Triggers SAA Aggregation and Accelerates Fibril Formation in the C20/A4 Alkaptonuria Cell Model
Source: Cells. 2024 Sep 7;13(17):1501. doi: 10.3390/cells13171501 (PMC11394027; doi:10.3390/cells13171501)

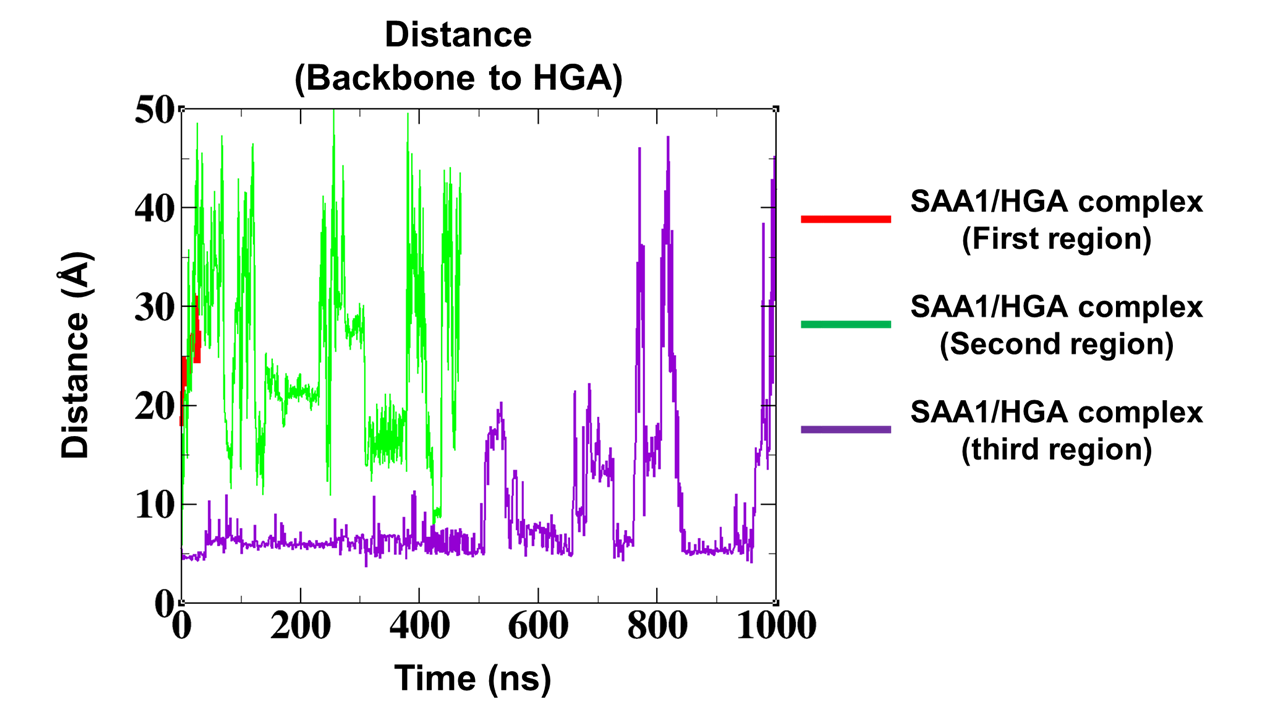

Supplement: Supplementary file 1 [file cells-13-01501-s001.zip › Figure S1.PNG]

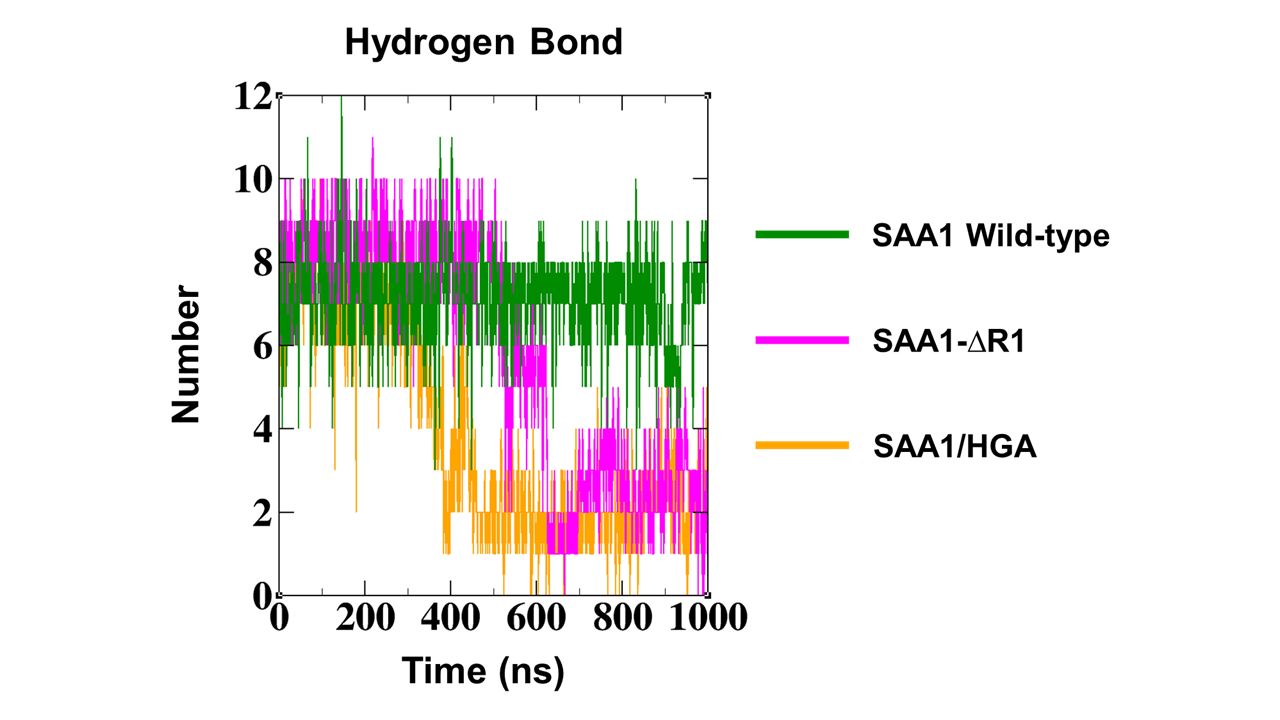

Supplement: Supplementary file 1 [file cells-13-01501-s001.zip › Figure S2.PNG]

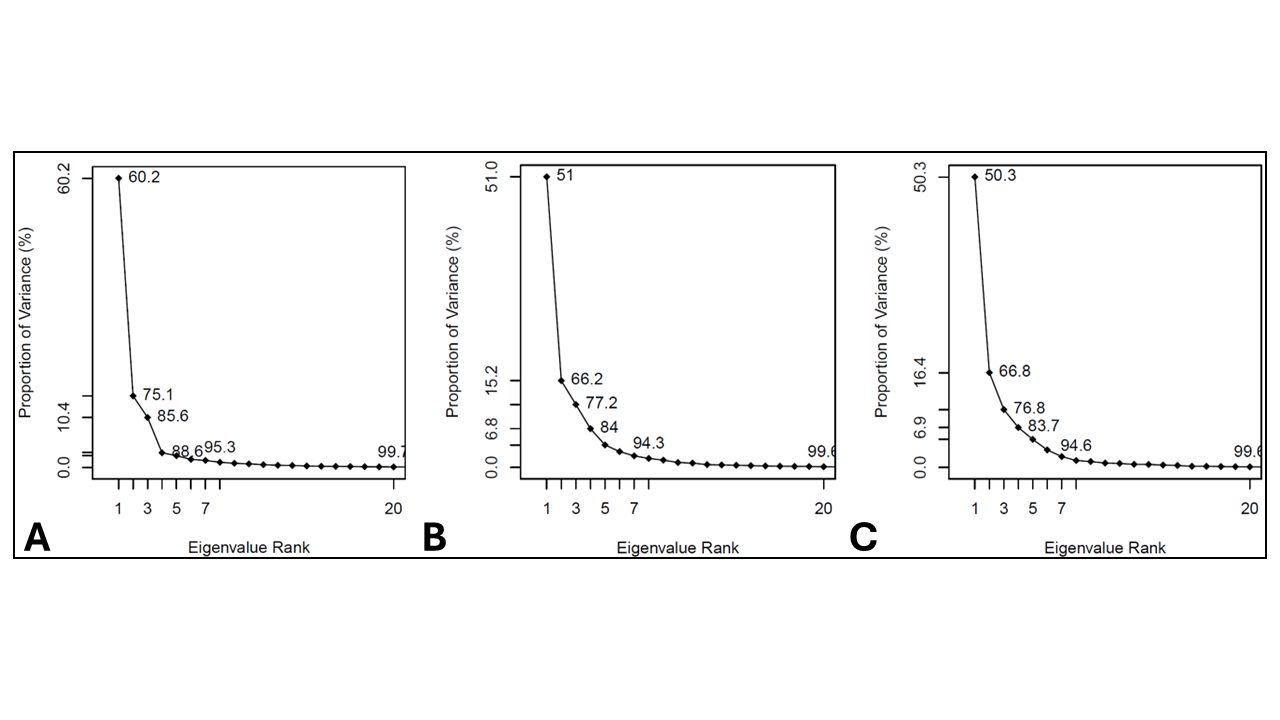

Supplement: Supplementary file 1 [file cells-13-01501-s001.zip › Figure S3.PNG]
